# Effects of Pregnancy on Plasma Sphingolipids Using a Metabolomic and Quantitative Analysis Approach

**DOI:** 10.3390/metabo13091026

**Published:** 2023-09-21

**Authors:** Luke F. Enthoven, Yuanyuan Shi, Emily Fay, Agnes Kim, Sue Moreni, Jennie Mao, Nina Isoherranen, Rheem A. Totah, Mary F. Hebert

**Affiliations:** 1 Department of Pharmacy, University of Washington, Seattle, WA 98195, USA; 2 Department of Medicinal Chemistry, University of Washington, Seattle, WA 98195, USArtotah@uw.edu (R.A.T.); 3 Department of Obstetrics and Gynecology, University of Washington, Seattle, WA 98195, USA; 4 Department of Pharmaceutics, University of Washington, Seattle, WA 98195, USA; ni2@uw.edu

**Keywords:** metabolomics, sphingolipids, ceramides, pregnant, postpartum, lipidome, sphingosines

## Abstract

Changes in the maternal metabolome, and specifically the maternal lipidome, that occur during pregnancy are relatively unknown. The objective of this investigation was to evaluate the effects of pregnancy on sphingolipid levels using metabolomics analysis followed by confirmational, targeted quantitative analysis. We focused on three subclasses of sphingolipids: ceramides, sphingomyelins, and sphingosines. Forty-seven pregnant women aged 18 to 50 years old participated in this study. Blood samples were collected on two study days for metabolomics analysis. The pregnancy samples were collected between 25 and 28 weeks of gestation and the postpartum study day samples were collected ≥3 months postpartum. Each participant served as their own control. These samples were analyzed using a Ultra-performance liquid chromatography/mass spectroscopy/mass spectroscopy (UPLC/MS/MS) assay that yielded semi-quantitative peak area values that were used to compare sphingolipid levels between pregnancy and postpartum. Following this lipidomic analysis, quantitative LC/MS/MS targeted/confirmatory analysis was performed on the same study samples. In the metabolomic analysis, 43 sphingolipid metabolites were identified and their levels were assessed using relative peak area values. These profiled sphingolipids fell into three categories: ceramides, sphingomyelins, and sphingosines. Of the 43 analytes measured, 35 were significantly different during pregnancy (*p* < 0.05) (including seven ceramides, 26 sphingomyelins, and two sphingosines) and 32 were significantly higher during pregnancy compared to postpartum. Following metabolomics, a separate quantitative analysis was performed and yielded quantified concentration values for 23 different sphingolipids, four of which were also detected in the metabolomics study. Quantitative analysis supported the metabolomics results with 17 of the 23 analytes measured found to be significantly different during pregnancy including 11 ceramides, four sphingomyelins, and two sphingosines. Fourteen of these were significantly higher during pregnancy. Our data suggest an overall increase in plasma sphingolipid concentrations with possible implications in endothelial function, gestational diabetes mellitus (GDM), intrahepatic cholestasis of pregnancy, and fetal development. This study provides evidence for alterations in maternal sphingolipid metabolism during pregnancy.

## 1. Introduction

During pregnancy, the maternal metabolome undergoes many changes to meet the physiologic demands imposed by a growing fetus and placenta [1]. The impact of these changes on maternal sphingolipid levels and sphingolipid metabolism in healthy pregnancies is still not determined. Sphingolipids are known to be involved in regulating cellular processes such as proliferation, apoptosis, and senescence, but their specific roles during pregnancy are not well defined [2]. Past studies have evaluated the levels of specific sphingolipids in various pregnant conditions, but none have used broad spectrum metabolomics coupled with targeted quantitative analysis to evaluate alterations in a range of sphingolipids in healthy pregnancies. Some of these lipids can be potential biomarkers for preeclampsia, gestational diabetes mellitus, and intrauterine growth restriction [3,4].

Human sphingolipid synthesis begins with de novo ceramide synthesis in the endoplasmic reticulum from palmitoyl-CoA and L-serine by the enzyme serine palmitoyl transferase (SPT) [1,3]. Ceramide can then be transformed in a variety of ways to form different sphingolipids with different unique properties and functions, including roles in endothelial function and blood glucose regulation [4,5]. This has led to an investigation into the impact of sphingolipids on gestational diabetes, intrahepatic cholestasis of pregnancy, preeclampsia, and intrauterine growth restriction where significant correlations have been reported between the levels of plasma sphingolipids and the development of pregnancy-related diseases [5,6,7]. Sphingolipids are secreted into the plasma with lipoproteins, therefore changes in plasma sphingolipids could reflect changes in sphingolipid synthesis or the availability of different lipoprotein species, among other contributing factors [8]. The objective of this analysis was to determine the effects of pregnancy on plasma sphingolipids. This was achieved by performing untargeted metabolomics on plasma samples during pregnancy as compared with postpartum samples. This was coupled with a targeted quantitative analysis to both confirm the metabolomics results and provide data for specific sphingolipids that were not identified in the metabolomics study. The dataset used for metabolomics has been shared in a previously published study [9]. 

## 2. Materials and Methods

### 2.1. Study Participants

All participants were between 18 and 50 years of age and were originally enrolled to participate in a larger study designed to evaluate the effects of retinoids on CYP2D6 activity [10]. All participants provided written informed consent. The study was approved by the institutional review board at the University of Washington and conducted in accordance with its guidelines. Study methods were the same as those used in our previous study as the data originated from the same samples [9]. 

Participants were recruited from the University of Washington’s hospitals and clinics. Participants were considered healthy when enrolled in the study and subject to a variety of health-based exclusion criteria including the use of certain medications detailed in the Supplementary Materials of our previous analysis [9]. Participants completed 3 study days with samples from study day 1 (SD1) during pregnancy and study day 3 (SD3) postpartum (≥3 months) used for this current analysis. Participants’ weight and height were recorded before each study day. A single 30 mL blood sample was collected from each participant on each study day in EDTA vacutainer tubes. Each sample had plasma separated by centrifugation (3000× *g* at 4 °C for 10 min) and frozen at −80 °C until analysis. Participants’ diet was recorded during the 3 days leading up to each study day and analyzed using a Student’s *t*-test between SD1 and SD3.

Participants also had blood samples drawn for analysis by the University of Washington Medical Center Department of Laboratory Medicine. These samples were used to report levels of albumin, bilirubin, serum creatinine, and blood urea nitrogen (BUN). This data was analyzed with a Student’s t-test between SD1 and SD3. Participants recorded all dietary intake, including food and beverages, on the three days leading up to each study day. Each participant’s daily lipid intake was then determined using Fooducate Ltd. (San Francisco, CA, USA).

### 2.2. Metabolomics Analysis

Metabolomics analysis was carried out by Metabolon Inc. (Morrisville, NC, USA) and adapted from previously described methods [11]. Metabolon performed all necessary preparation, analysis, and quality control for all samples. Extracts from samples were analyzed with reverse-phase ultra-performance liquid chromatography with tandem mass spectrometry (UPLC-MS/MS) using both positive and negative electrospray ionization. 

A global biochemical profiling analysis was used to analyze the extracted plasma samples and employed four unique columns that are all detailed in our previous paper [8]. The first UPLC run was a C18 column (Waters UPLC BEH C18-2.1 × 100 mm, 1.7 µm) (Waters Corporation, Milford, MA, USA) with a 0.35 mL/min flow rate and water (mobile phase A) and methanol (mobile phase B) mobile phases optimized for hydrophilic compounds (0.05% perfluoropentanoic acid (PFPA) and 0.1% formic acid, pH~2.5, LC/MS Positive ionization). LC/MS Pos Polar utilized a linear gradient from 5% mobile phase B to 80% mobile phase B over 3.35 min.

The second UPLC run utilized a Waters C18 column with a 0.60 mL/min flow rate and water (mobile phase C) and 50% methanol with 50% acetonitrile (mobile phase D) mobile phases optimized for hydrophobic compounds (0.05% PFPA and 0.1% formic acid, pH~2.5, LC/MS Pos Lipid). This was the method used for the profiling of all identified sphingolipids. LC/MS Pos Lipid utilized a linear gradient from 40% mobile phase D to 99.5% mobile phase D over 1 min, holding 99.5% phase D for 2.4 min.

The third UPLC run utilized the same Waters C18 column with a 0.35 mL/min flow rate, this time including water (mobile phase E) and 95% methanol with 5% water (mobile phase F) mobile phases (6.5 mM ammonium bicarbonate), pH 8, LC/MS Negative ionization). LC/MS Neg utilized a linear gradient from 0.5% mobile phase F to 70% mobile phase F over 4 min followed by a rapid gradient to 99% mobile phase F in 0.5 min.

The fourth UPLC run used a different column specific for hydrophobic interactions chromatography (Waters UPLC BEH Amide 2.1 × 150 mm, 1.7 µm) with a 0.5 mL/min flow rate, this time with 15% water, 5% methanol, and 80% acetonitrile (mobile phase G) and 50% water and 50% acetonitrile (mobile phase H) mobile phases (10 mM ammonium formate, pH 10.8, LC/MS Polar). LC/MS Polar utilized a linear gradient from 5% mobile phase H to 50% mobile phase H over 3.5 min followed by a linear gradient from 50% mobile phase H to 95% mobile phase H in 2 min.

For all methods, the MS analysis alternated between full scan MS and data-dependent MS*^n^* scans, generally covering 70–1000 *m*/*z*. An AB Sciex 6500plus mass spectrometer was used. Features in the experimental samples were automatically compared to a reference library and curated for quality assurance by software (AB Sciex, Toronto, Ontario, Canada) developed at Metabolon Inc. Quality control and data curation were performed in order to ensure that the identification of metabolites was consistent and true. Data identified as background noise and system artifacts were removed. Peak identification was also performed using Metabolon Inc.-developed software. Quantification of peaks was performed using area-under-the-curve obtained from the MS/MS raw peak data. Analysis spanned multiple days and required normalization. All peak areas were normalized to a median of one (1.00) for every individual metabolite. This was done to correct for day-to-day instrumental variation. 

### 2.3. Targeted Quantitative Analysis

Targeted quantitative analysis focused on quantifying sphingolipids in the plasma samples. This was completed for the sphingolipids for which standards were commercially available and to compare with the results of the metabolomics analysis. Plasma samples used were the same as those whose collection is referred to in Section 2.1. The samples were analyzed as previously described [12].

First, lipids were extracted using an organic protein precipitation solvent mixture of methyl tert-butyl ether (50%), methanol (40%), and isopropanol (10%). The precipitation solvent mixture was spiked with 20 µL of internal standard (Ceramide/Sphingoid Internal Standard Mixture I, 25 µM, Avanti Polar Lipids, LM6002) yielding a final standard concentration of 19.4 nM. 

Ten µL of each plasma sample were transferred to a Masterblock; Greiner Bio-One 96-deep well polypropylene microtiter plate. Then 190 µL of the previously described precipitation solvent was added to each well and the plate was sealed with a MicroLiter cap mat with a Wheaton PTFE (DWK Life Sciences, Millville, NJ, USA) barrier applied to it. The plate was then mixed on a multitube vortex at speed 10 for 5 min. A 10 µm Captiva; Agilent filter plate was placed above a new Masterblock plate. The samples were transferred to the second filter plate, with about 50 µL flowing through. The first filter plate was discarded. 

For each sample on the new plate, 450 µL of a 65% methanol and 25% isopropanol (v:v) mixture was added. The plate was then sealed and 5 µL of the solution in each well was injected with an autosampler while cooled to 8 °C and resolved using reverse-phase chromatography.

With the column oven heated to 50 °C, samples were run on an Acquity UPLC Protein BEH C4 Column, 300 Å, 1.7 µm, 2.1 mm × 50 mm analytical column with an Acquity UPLC Protein BEH C4 VanGuard Pre-column, 300 Å, 1.7 µm, 2.1 mm × 5 mm guard column. Mobile phase A included water with 0.2% formic acid and mobile phase B included 60% acetonitrile, 40% isopropanol, and 0.2% formic acid. A linear gradient of 49% to 79% of mobile phase B, over 8.4 min at 0.4 mL/min, was used to elute analytes from the column. These analytes were then detected using an AB Sciex 6500plus mass spectrometer (AB Sciex, Toronto, Ontario, Canada) with parameters optimized for each compound. The table of collision energies and declustering potentials for each analyte are available in a previous paper [12].

Internal standards were included in the precipitation solvent to control for methodological variability. The standard was the ceramide/sphingolipid internal standard mixture I, 25 μmol/L; Avanti Polar Lipids, LM-6002 present at a concentration of 19.4 nmol/L that included Ceramide (d18:1/12:0), Ceramide (d18:1/25:0), Glucosylceramide (d18:1/12:0), Lactosylceramide (d18:1/12:0), and Sphingomyelin (d18:1/12:0) (Avanti Polar Lipids Inc., Alabaster, AL, USA). Skyline software was used to determine chromatographic peak areas [13]. The peak area ratio for each eluted sphingolipid was the peak area of the eluted endogenous analyte from a single MS/MS transition divided by the sum of the peak area of five internal standards. This ratio for each analyte was then divided by the mean peak area ratio of the single-point calibrator. More information on the calibration and determination of lipid concentrations is available in a previous paper [12].

### 2.4. Statistical Analysis

A paired Student’s *t*-test was used to compare pregnant and postpartum groups. The R Studio default paired *t*-test function was used to perform these tests. The same *t*-test function was also used to determine significant differences between lipid intake on the two study days. 

Pregnant/postpartum ratios were calculated using the mean of the ratio of each individual pregnant and postpartum data pair. The *p*-values for all metabolites were determined and used to rank the metabolites from lowest to highest *p*-value. We then used a q-value to estimate the type one error rate for the hundreds of comparisons performed. All metabolites had a q-value generated using the BioConductor q-value function in R Studio. This function uses *p*-values and *p*-value ranking to determine q-values where $Q_{j}=Q\left(P_{j}\right)=minFDR{\left(t\right)}_{t\geq P_{j}}$ [13,14]. FDR refers to the ratio of false positives to total positives. We used a significance cutoff of *p* < 0.05 and q < 0.01.

Quantitative concentration data was also analyzed using a paired Student’s *t*-test. *p*-values were transformed using a Bonferroni correction to account for the 23 quantitative comparisons. A *p*-value of <0.05/23 i.e., <0.002 was considered significant. Pregnant/postpartum ratio values were calculated using the mean of the ratios for each individual data pairing. 

### 2.5. Sphingolipid Naming Conventions

Sphingolipids are denoted using generally accepted naming conventions. This resulted in sphingolipids being denoted as A (nX:Y/C:D) where A is the family name for the compound which incorporates a name for the head group if one is present; n represents the number of OH groups with a d indicating two OH groups; X is the number of carbons in the sphingoid backbone; Y is the saturation of the sphingoid back bone; C is the number of carbons in the fatty acid chain; and D is the saturation of the fatty acid chain.

## 3. Results

### 3.1. Characteristics of Subjects

Although 81 subjects were enrolled in the main study, only the 47 women who completed both study days were included in this analysis. Their races/ethnicities were as follows: 74% White (*n* = 35), 9% Black (*n* = 4), 13% Asian (*n* = 6), 2% Hispanic (*n* = 1), and 2% Pacific Islander (*n* = 1). Subject demographics and laboratory tests can be found in Table 1. Samples were collected on three study days as part of a larger study evaluating the effects of retinoids on cytochrome P450 and 2D6 (CYP2D6) activity during pregnancy and postpartum, but samples from only two of those days were subject to metabolomics analysis during pregnancy (25–28 weeks gestation) and postpartum (≥3 months). 

As expected, patient weight was significantly higher during pregnancy compared to postpartum (*p* < 0.05). Total daily dietary lipid intake was higher during pregnancy than ≥3 months postpartum (86.3 ± 36.7 g vs. 70.2 ± 25.9 g, *p* < 0.03). Serum albumin, bilirubin, creatinine, and blood urea nitrogen were all significantly lower during pregnancy than postpartum (*p* < 0.05). Measured creatinine clearance was significantly higher during pregnancy than postpartum (*p* < 0.05).

Metabolomics analysis identified 980 unique metabolites in the samples, 706 of which were determined to be significantly different between pregnant and postpartum study days (data for all identified metabolites can be found in Supplementary File S1). Quantitative analysis was then performed, yielding concentration values for 23 sphingolipids which can be found in Table 2. Four of the sphingolipids measured in quantitative analysis were also analyzed in metabolomics. Seventeen of the 23 sphingolipids were found to have significantly different concentrations between pregnant and postpartum conditions.

### 3.2. Ceramide, Sphingolipid, and Sphingosine

**Figure 1:**
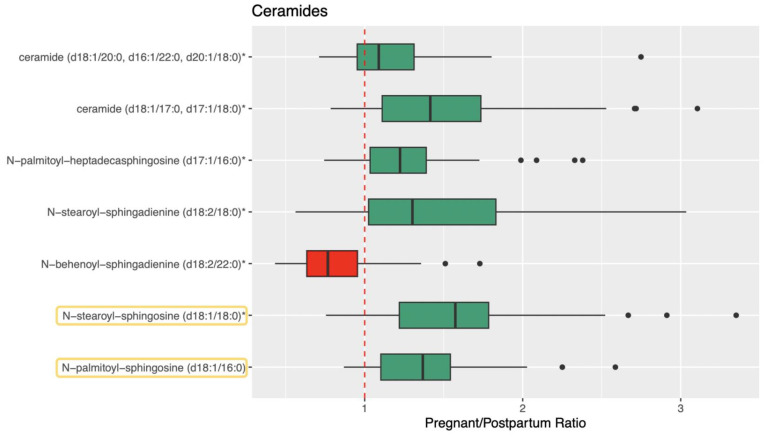
Pregnant/Postpartum ratios for ceramide metabolites. Ratios are calculated using normalized relative peak area from metabolomics analysis. Ratios greater than 1 are shown as green boxes and ratios less than 1 are shown as red boxes. Quartiles are shown as the upper and lower bounds of each box with the median in the middle. The whiskers are equal to the maximum value minus 1.5 times the interquartile range and the minimum value plus 1.5 times the interquartile range. Points beyond the whiskers are outliers. A ratio value of 1 is shown with a red dashed line. Confirmed analytes are shown circled in yellow. The single asterisk (*) indicates metabolites that were annotate based on silico phase I prediction.

**Figure 2:**
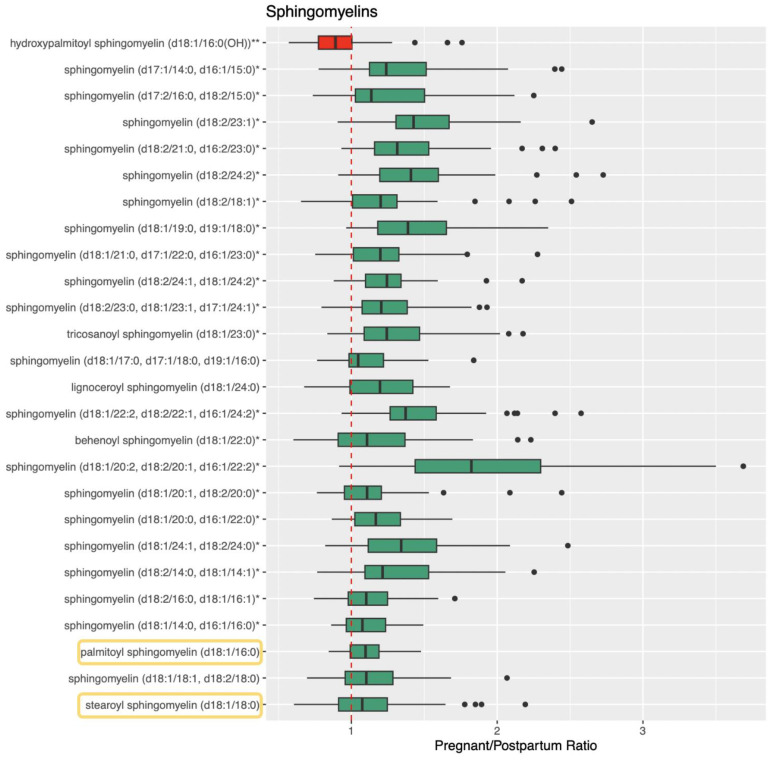
Pregnant/Postpartum ratios for sphingomyelin metabolites. Ratios are calculated using normalized relative peak area from metabolomics analysis. Ratios greater than 1 are shown as green boxes and ratios less than 1 are shown as red boxes. Quartiles are shown as the upper and lower bounds of each box with the median in the middle. The whiskers are equal to the maximum value minus 1.5 times the interquartile range and the minimum value plus 1.5 times the interquartile range. Points beyond the whiskers are outliers. A ratio value of 1 is shown with a red dashed line. Confirmed analytes are shown circled in yellow. The single asterisk (*) indicates metabolites that were annotate based on silico phase I prediction. The double asterisk (**) indicates metabolites that were annotate based on silico phase II prediction.

**Figure 3:**
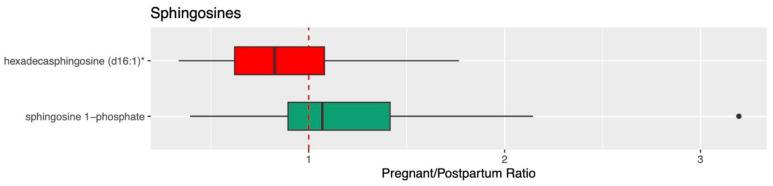
Pregnant/Postpartum ratios for sphingosine metabolites. Ratios are calculated using normalized relative peak area from metabolomics analysis. Ratios greater than 1 are shown as green boxes and ratios less than 1 are shown as red boxes. Quartiles are shown as the upper and lower bounds of each box with the median in the middle. The whiskers are equal to the maximum value minus 1.5 times the interquartile range and the minimum value plus 1.5 times the interquartile range. Points beyond the whiskers are outliers. A ratio value of 1 is shown with a red dashed line. Confirmed analytes are shown circled in yellow. The single asterisk (*) indicates metabolites that were annotate based on silico phase I prediction.

Figure 1, Figure 2 and Figure 3 visualize the mean normalized peak area data for ceramide, sphingolipid, and sphingosine metabolites and the results of the direct comparison of the metabolite peak areas between pregnancy and postpartum (represented by pregnancy/postpartum ratios). Data tables containing these values can be found in Supplementary File S2. Forty-three metabolites involved in the metabolism of ceramides, sphingomyelins, and sphingosines were identified and 35 were found to be significantly different during pregnancy compared to postpartum based on the metabolomics analysis. Of these, 32 were significantly higher and three were significantly lower during pregnancy. 

**Figure 4:**
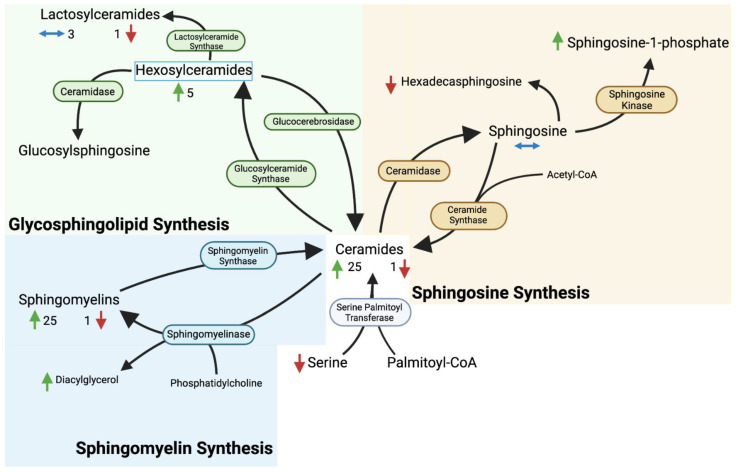
The metabolic relationship between the 3 types of sphingolipids discussed in this paper. The blue enzymes and background represent parts of sphingomyelin synthesis; the yellow enzymes and background represent sphingosine synthesis; and the green enzymes and background represent glycosphingolipid synthesis. All are synthesized from ceramides. Green arrows represent an increase during pregnancy, red arrows represent a decrease during pregnancy, and horizontal blue arrows represent no significant change during pregnancy. The categorical metabolites (ceramides and sphingomyelins) are shown with numbers representing how many of the metabolites in those categories were higher or lower during pregnancy.

For the ceramides, 11 metabolites were identified and seven were significantly different during pregnancy compared to postpartum (Figure 1). For the sphingomyelins, 29 metabolites were identified and 26 were significantly different during pregnancy (Figure 2). For the sphingosines, three metabolites were identified and two were significantly different during pregnancy (Figure 3). All three of these sphingolipid groups are shown in metabolic relation to each other in Figure 4. The most altered metabolite of interest in metabolomics was sphingomyelin (d18:1/20:2, d18:2/20:1, d16:1/22:2) with a pregnancy/postpartum value of 1.83, as shown in Figure 2. 

Quantitative, targeted analysis was completed for 23 sphingolipids; 17 ceramides and six sphingomyelins. Of these 23 analytes, four were also detected in the metabolomics analysis. Seventeen of the 23 compounds measured had significantly different concentrations between pregnancy and postpartum with 13 ceramides and four sphingomyelins. Fourteen were significantly higher, three significantly lower, and six were not significantly altered during pregnancy. The four analytes measured both in metabolomics analysis and quantitative analysis were all confirmed to be significantly different and all matched pregnant/postpartum ratios between the same compounds in both analyses (1.10 vs. 1.16 for palmitoyl sphingomyelin (d18:1/16:0), 1.13 vs. 1.24 for stearoyl sphingomyelin (d18:1/18:0), 1.39 vs. 1.26 for N-palmitoyl-sphingosine (d18:1/16:0), and 1.58 vs. 1.53 for N-stearoyl-sphingosine (d18:1/18:0)).

## 4. Discussion

### 4.1. Overview

Several studies have investigated the impact of specific diseases during pregnancy on the plasma concentrations of ceramides, sphingomyelins, and sphingosines [3,4,5,6,7], but none have reported the difference in plasma concentrations of these lipids between pregnancy and postpartum in healthy pregnancies. Past research has explored levels of broad categories of sphingolipids such as sphingomyelin 18:1 or sphingomyelin 24:0 in preeclampsia and gestational diabetes [15]. These studies focused on changes in broad categories of sphingolipids and do not report levels of individual sphingolipids [5,15]. Other studies focused on a small number of specific sphingolipids with the potential to serve as biomarkers for preeclampsia such as spingosine-1-phosphate (S1P) [7]. This study is the first to evaluate differences in the levels of reported ceramide and sphingomyelin compounds except for behenoyl sphingomyelin (d18:1/22:0), stearoyl sphingomyelin (d18:1/18:0), and palmitoyl sphingomyelin (d18:1/16:0) which have been previously reported [16]. Our study is unique in combining untargeted metabolomics with targeted quantitative analysis, between pregnant and postpartum conditions, with subjects serving as their own controls. We are also the first to report levels of *N*-behenoyl-sphingadienine (d18:2/22:00), hydroxypalmitoyl sphingomyelin (d18:1/16:0(OH)), and hexadecasphingosine (d16:1) changes during pregnancy. Little is known about the function of these three sphingolipids and it remains unknown why they decreased during pregnancy in our cohort. At a minimum, our findings provide a basis for further research to better understand the response of sphingolipid levels to pregnancy and serve as a normal comparison in the continuing effort to understand the role of sphingolipids in pregnancy-related diseases.

Our analysis indicates significant changes in ceramide and sphingomyelin levels during pregnancy. The sphingolipids measured were found to have pregnant/postpartum ratios between 0.82 and 1.94, with all but six (Ceramide (d18:1/24:0), Ceramide (d18:1/22:0), Lactosylceramide (d18:1/24:0), hydroxypalmitoyl sphingomyelin, hexadecasphingosine (d16:1), and N-behenoyl-sphingadienine (d18:2/22:0)) being significantly higher during pregnancy in metabolomics and quantitative analysis. This indicates a relatively consistent, mild-to-moderate increase in these sphingolipid metabolites during pregnancy.

A specific sphingosine metabolite, S1P, has been previously identified as a biomarker associated with pregnancy-related diseases and disorders. Plasma S1P was reported to be higher in normal pregnancy than in non-pregnant controls, consistent with our data. Further increases in plasma S1P are associated with preeclampsia, gestational diabetes, and intrauterine growth restriction [17,18].

Ceramide (d16:1/24:1, d18:1/22:1) and ceramide (d18:2/24:1, d18:1/24:2), are also associated with gestational diabetes mellitus and have the potential to serve as biomarkers for the disease. Gestational diabetes has been correlated with decreased levels of these 24:1 ceramides [18,19]. We did not observe these ceramides to be significantly altered in our cohort of healthy pregnant subjects.

The impact of sphingosine 1-phosphate and ceramides on endothelial function and vasodilation have been evaluated and found to be critical for a healthy pregnancy [20,21]. Lipids, such as S1P mediate the endothelial effects of high-density lipoprotein (HDL). This effect appears to be the result of changes in the expression of endothelial nitric oxide synthase resulting from interaction between certain sphingolipids (including S1P) and the lysophospholipid receptor S1P3, mimicking the already well-documented vasoactive effects of HDL [22]. Changes in expression of SIP3 result in changes in nitric oxide production and consequently impact endothelial function through the production of endothelial nitric oxide [23]. This aligns with the decrease in plasma levels of nitric oxide and related metabolites that has been observed in healthy pregnancies by Ziegler et al. [24]. Both sphingosine 1-phosphate and ceramide concentrations in plasma are higher during pregnancy and have been connected to changes in nitric oxide metabolism. This could reflect an endothelial protective function of sphingolipids during pregnancy through the mediation of vasodilation to preserve endothelial functioning throughout pregnancy [7,23,24,25].

The relationship between plasma concentrations of sphingolipids and intrahepatic cholestasis of pregnancy (ICP) has also been evaluated by Sun et al. [26] We observed an almost ubiquitous increase in sphingolipids during pregnancy while Sun et al. observed both increases and decreases in different sphingolipids, including both ceramides and sphingomyelins. It is possible that ICP pathology is related to a metabolic inability to produce a high enough concentration of sphingolipids during pregnancy but no specific conclusions can be made at this time about the mechanism by which sphingolipids modulate ICP [26]. 

In another study evaluating plasma lipidomics in patients with gestational diabetes mellitus, higher concentrations of sphingolipids were associated with a lower risk of gestational diabetes [16]. The broad elevation in sphingolipids we observed during pregnancy might offer some gestational diabetes mellitus protection in that gestational diabetes mellitus could be related to an inability to produce the higher concentrations of plasma sphingolipids we observed in healthy pregnancies, but more research is needed to define the association of plasma sphingolipids with gestational diabetes [16].

### 4.2. Limitations

The use of only untargeted semi-quantitative metabolomic analysis for a majority of analytes limits the specific conclusions that can be drawn from this study. However, univariate analysis combined with quantitative analysis yielded strong evidence for an increase in plasma sphingolipid levels during pregnancy. This study is also limited by the single time point during pregnancy and postpartum that samples were collected and only reflects the levels measured at these specific time points. In addition, total dietary fat intake was significantly higher during pregnancy than postpartum. Although study samples were collected after a meal, there is no evidence that plasma sphingolipids are altered postprandially [27]. Quantitative analysis was limited by the availability of sphingolipid analytical standards. This prevented us from directly confirming more sphingolipid species from the metabolomics analysis. Future studies that collect samples serially across pregnancy and postpartum will be able to establish time courses for sphingolipid concentrations. 

### 4.3. Conclusions

We have demonstrated that a wide range of sphingolipids have altered plasma concentrations during pregnancy compared to postpartum. This includes ceramides, sphingomyelins, and sphingosines. Such alterations align with the higher levels found in previously published work exploring sphingolipids during pregnancy and pregnancy-related diseases. This is the first investigation to report both semi-quantitative and quantitative plasma levels of specific sphingolipids as opposed to categorical levels. Of the measured analytes in the metabolomics analysis, a large majority were higher during pregnancy compared to the postpartum state, which was consistent with subsequent quantitative analysis. Such changes might reflect alterations of maternal metabolism and dietary intake during pregnancy. These physiological changes in the lipidome during pregnancy have possible implications in the pathologies of ICP, GDM, and preeclampsia. This use of metabolomics analysis with targeted quantitative analysis has been used to draw broad conclusions and generate novel hypotheses surrounding sphingolipids that provide a baseline comparison for sphingolipids in healthy pregnancies. These findings can also serve as a point of comparison in the search for biomarkers of pregnancy-related diseases. More research is needed to determine the exact role of sphingolipid changes during pregnancy and to understand the causes of the increase in plasma sphingolipids in the pregnant state.

## Figures and Tables

**Table 1:** Characteristics of Subjects During Pregnancy and Postpartum.

Characteristics	Pregnant (n = 47)	Postpartum (n = 47)	*p*-Value
Gestational Age (weeks) or Time Postpartum (weeks)	27.0 ± 1.3	15.0 ± 2.1	NA
Height (cm)	163.6 ± 16.8		NA
Weight (kg)	71.6 ± 10.6	67.4 ± 9.3	1 × 10^−5^
IBW (kg)		58.2 ± 6.4	NA
BMI (kg/m^2^)		25.7 ± 3.2	NA
Albumin (g/dL)	3.6 ± 0.2	4.6 ± 0.2	9 × 10^−28^
Bilirubin (mg/dL)	0.5 ± 0.2	0.7 ± 0.4	7 × 10^−7^
Serum Creatinine (mg/dL)	0.5 ± 0.1	0.7 ± 0.1	9 × 10^−21^
BUN (mg/dL)	7.8 ± 1.9	14.0 ± 4.0	1 × 10^−14^
CrCl (mL/min)	194.3 ± 81.4	130.9 ± 25.6	3 × 10^−6^

*p*-values from paired Student’s *t*-test. BMI calculated using pre-pregnancy body weight. BMI = body mass index. IBW = ideal body weight. BUN = blood urea nitrogen. CrCl = creatinine clearance.

**Table 2:** Concentrations of the Quantified Sphingosines, Ceramides, and Sphingomyelins During Pregnancy and Postpartum.

Sphingolipid	Pregnant (nM)	Postpartum (nM)	Pregnancy/ Postpartum	Corrected *p*-Value
HexCer (d18:1/16:0)	120 ± 20	80 ± 20	1.56 ± 0.28	8.08 × 10^−17^
Lactosylceramide (d18:1/16:0)	540 ± 120	350 ± 70	1.54 ± 0.28	6.85 × 10^−16^
HexCer (d18:1/20:0)	17.7 ± 5	11.6 ± 3	1.57 ± 0.34	8.19 × 10^−14^
HexCer (d18:1/18:0)	16.9 ± 5	10.4 ± 3	1.68 ± 0.48	6.45 × 10^−12^
HexCer (d18:1/22:0)	140 ± 30	110 ± 20	1.40 ± 0.31	8.88 × 10^−10^
Ceramide (d18:1/14:0)	7.04 ± 2	5.06 ± 1	1.45 ± 0.41	2.44 × 10^−9^
Palmitoyl sphingomyelin (d18:1/16:0)	128,000 ± 15,800	111,000 ± 16,400	1.16 ± 0.14	2.00 × 10^−8^
HexCer (d18:1/24:0)	140 ± 30	110 ± 30	1.27 ± 0.24	9.92 × 10^−8^
Stearoyl sphingomyelin (d18:1/18:0)	4290 ± 780	3510 ± 670	1.24 ± 0.22	1.66 × 10^−7^
Ceramide (d18:1/24:0)	2300 ± 570	3030 ± 710	0.79 ± 0.21	3.84 × 10^−7^
Sphingomyelin (d18:1/20:0)	2270 ± 380	1880 ± 350	1.23 ± 0.21	4.01 × 10^−7^
N-palmitoyl-sphingosine (d18:1/16:0)	230 ± 40	190 ± 40	1.26 ± 0.26	1.19 × 10^−6^
Ceramide (d18:1/20:0)	60 ± 10	46.2 ± 10	1.37 ± 0.34	1.72 × 10^−6^
N-stearoyl-sphingosine (d18:1/18:0)	64 ± 20	45.7 ± 20	1.53 ± 0.52	1.76 × 10^−6^
Sphingomyelin (d18:1/14:0)	5300 ± 1230	4540 ± 1020	1.19 ± 0.22	4.99 × 10^−5^
Ceramide (d18:1/22:0)	400 ± 90	470 ± 140	0.91 ± 0.25	2.47 × 10^−2^
Lactosylceramide (d18:1/24:0)	66.3 ± 10	74.8 ± 20	0.91 ± 0.19	4.57 × 10^−2^
Ceramide (d18:1/24:1)	250 ± 50	220 ± 60	1.16 ± 0.28	7.51 × 10^−2^
Sphingomyelin (d18:1/22:0)	3150 ± 550	2880 ± 600	1.12 ± 0.21	0.207
Lactosylceramide (d18:1/18:0)	9.74 ± 3	9.03 ± 2	1.11 ± 0.27	0.769
Sphingomyelin (d18:1/24:0)	17,100 ± 3100	16,000 ± 3340	1.09 ± 0.21	1
Lactosylceramide (d18:1/20:0)	2.80 ± 1	25.6 ± 10	1.16 ± 0.41	1
Lactosylceramide (d18:1/22:0)	10.2 ± 3	10.7 ± 3	1.00 ± 0.34	1

Pregnant and postpartum values represent concentrations. *p* values from paired Student’s *t*-test. *p*-values have undergone Bonferroni correction to account for multiple comparisons. Hexosylceramide is abbreviated to HexCer. All data generated for quantitative analysis can be found in Supplementary File S1.

## Data Availability

The metabolomics data generated for this study is available in the Supplementary Materials as a .csv file.

## Supplementary Materials

The following are available online at https://www.mdpi.com/article/10.3390/metabo13091026/s1, XLSX File S1: Quantitative Analysis Data and DOCX File S2: Sphingolipid Metabolomics Data Tables. All other data can be found in the Supplementary Files of our previous paper [9].
