# Supplementary material for: Effects of Pregnancy on Plasma Sphingolipids Using a Metabolomic and Quantitative Analysis Approach
Source: Metabolites. 2023 Sep 21;13(9):1026. doi: 10.3390/metabo13091026 (PMC10534641; doi:10.3390/metabo13091026)
Supplement: Supplementary file 1 [file metabolites-13-01026-s001.zip › Sphingolipid Metabolomics Data Tables.pdf]

Table 1. Ceramide Pregnant and Postpartum Metabolite Batch Normalized and Imputed Peak Areas.

Ceramides	Pregnant	Postpartum	Pregnancy/ Postpartum	p-value	q-value
N-behenoyl-sphingadienine (d18:2/22:0)*	0.91 ± 0.25	1.19 ± 0.41	0.82 ± 0.26	9×10^{-7}	2×10^{-7}
ceramide (d18:1/20:0, d16:1/22:0, d20:1/18:0)*	1.07 ± 0.25	0.98 ± 0.25	1.15 ± 0.35	9×10^{-3}	1×10^{-3}
N-palmitoyl-heptadecasphingosine (d17:1/16:0)*	1.12 ± 0.33	0.91 ± 0.26	1.28 ± 0.36	1×10^{-6}	3×10^{-7}
N-palmitoyl-sphingosine (d18:1/16:0)	1.18 ± 0.26	0.88 ± 0.19	1.39 ± 0.37	3×10^{-10}	1×10^{-10}
ceramide (d18:1/17:0, d17:1/18:0)*	1.21 ± 0.39	0.84 ± 0.28	1.52 ± 0.55	3×10^{-9}	1×10^{-9}
N-stearoyl-sphingadienine (d18:2/18:0)*	1.24 ± 0.43	0.90 ± 0.31	1.49 ± 0.60	2×10^{-6}	5×10^{-7}
N-stearoyl-sphingosine (d18:1/18:0)*	1.27 ± 0.41	0.85 ± 0.27	1.58 ± 0.57	3×10^{-10}	1×10^{-10}

[1] Pregnant and postpartum values represent median scaled peak areas. P values from paired Student's t-test. P-values, q-values, and fold change for all identified metabolites can be found in the supplementary files of our previous paper [9].

Table 2. Sphingomyelins Pregnant and Postpartum Batch Normalized and Imputed Metabolite Peak Areas.

Sphingomyelins	Pregnant	Postpartum	Pregnancy/ Postpartum	p-value	q-value
hydroxypalmitoyl sphingomyelin (d18:1/16:0(OH))**	1.02 ± 0.29	1.13 ± 0.34	0.93 ± 0.25	6×10^{-3}	9×10^{-4}
sphingomyelin (d18:1/14:0, d16:1/16:0)*	1.05 ± 0.19	0.95 ± 0.18	1.11 ± 0.16	5×10^{-5}	1×10^{-5}
palmitoyl sphingomyelin (d18:1/16:0)	1.05 ± 0.13	0.97 ± 0.13	1.10 ± 0.14	4×10^{-5}	9×10^{-6}
stearoyl sphingomyelin (d18:1/18:0)	1.06 ± 0.22	0.99 ± 0.27	1.13 ± 0.33	0.03	4×10^{-3}
sphingomyelin (d18:1/20:1, d18:2/20:0)*	1.07 ± 0.24	0.97 ± 0.25	1.15 ± 0.32	5×10^{-3}	7×10^{-4}
sphingomyelin (d18:1/17:0, d17:1/18:0, d19:1/16:0)	1.07 ± 0.20	0.98 ± 0.20	1.11 ± 0.22	3×10^{-3}	4×10^{-4}
sphingomyelin (d18:1/20:0, d16:1/22:0)*	1.08 ± 0.16	0.92 ± 0.16	1.20 ± 0.23	2×10^{-7}	5×10^{-8}
sphingomyelin (d18:1/21:0, d17:1/22:0, d16:1/23:0)*	1.08 ± 0.23	0.91 ± 0.21	1.22 ± 0.29	2×10^{-6}	5×10^{-7}
sphingomyelin (d18:2/16:0, d18:1/16:1)*	1.09 ± 0.18	0.98 ± 0.15	1.13 ± 0.21	2×10^{-4}	3×10^{-5}
sphingomyelin (d18:1/18:1, d18:2/18:0)	1.10 ± 0.20	0.98 ± 0.20	1.15 ± 0.26	7×10^{-4}	1×10^{-4}
lignoceroyl sphingomyelin (d18:1/24:0)	1.10 ± 0.25	0.94 ± 0.18	1.19 ± 0.26	3×10^{-5}	6×10^{-6}
sphingomyelin (d18:2/24:1, d18:1/24:2)*	1.11 ± 0.18	0.90 ± 0.16	1.26 ± 0.25	3×10^{-5}	1×10^{-10}

behenoyl sphingomyelin (d18:1/22:0)*	1.11 ± 0.30	0.98 ± 0.23	1.18 ± 0.38	0.02	$2×10^{-3}$
sphingomyelin (d18:2/18:1)*	1.14 ± 0.26	0.95 ± 0.21	1.23 ± 0.36	$3×10^{-5}$	$7×10^{-6}$
sphingomyelin (d18:2/23:0, d18:1/23:1, d17:1/24:1)*	1.14 ± 0.23	0.92 ± 0.16	1.27 ± 0.27	$5×10^{-9}$	$2×10^{-9}$
tricosanoyl sphingomyelin (d18:1/23:0)*	1.16 ± 0.23	0.92 ± 0.15	1.29 ± 0.30	$3×10^{-9}$	$1×10^{-9}$
sphingomyelin (d18:2/14:0, d18:1/14:1)*	1.16 ± 0.38	0.90 ± 0.26	1.32 ± 0.33	$6×10^{-9}$	$2×10^{-9}$
sphingomyelin (d18:1/24:1, d18:2/24:0)*	1.17 ± 0.24	0.88 ± 0.19	1.37 ± 0.35	$1×10^{-9}$	$4×10^{-10}$
sphingomyelin (d18:2/24:2)*	1.18 ± 0.30	0.84 ± 0.22	1.45 ± 0.38	$1×10^{-12}$	$8×10^{-13}$
sphingomyelin (d17:2/16:0, d18:2/15:0)*	1.18 ± 0.42	0.95 ± 0.27	1.27 ± 0.37	$8×10^{-6}$	$2×10^{-6}$
sphingomyelin (d18:1/22:2, d18:2/22:1, d16:1/24:2)*	1.20 ± 0.26	0.84 ± 0.21	1.47 ± 0.37	$7×10^{-14}$	$5×10^{-14}$
sphingomyelin (d18:2/21:0, d16:2/23:0)*	1.21 ± 0.30	0.88 ± 0.18	1.40 ± 0.34	$3×10^{-12}$	$1×10^{-12}$
sphingomyelin (d17:1/14:0, d16:1/15:0)*	1.21 ± 0.40	0.91 ± 0.28	1.36 ± 0.39	$2×10^{-8}$	$7×10^{-9}$
sphingomyelin (d18:1/19:0, d19:1/18:0)*	1.21 ± 0.26	0.85 ± 0.19	1.47 ± 0.37	$2×10^{-13}$	$1×10^{-13}$
sphingomyelin (d18:2/23:1)*	1.22 ± 0.28	0.83 ± 0.17	1.49 ± 0.35	$3×10^{-15}$	$2×10^{-15}$
sphingomyelin (d18:1/20:2, d18:2/20:1, d16:1/22:2)*	1.41 ± 0.37	0.77 ± 0.22	1.94 ± 0.66	$3×10^{-16}$	$4×10^{-16}$

[1] Pregnant and postpartum values represent median scaled peak areas. P values from paired Student's t-test. P-values, q-values, and fold change for all identified metabolites can be found in the supplementary files of our previous paper [9].

Table 3. Sphingosines Pregnant and Postpartum Batch Normalized and Imputed Metabolite Peak Area.

Sphingosines	Pregnant	Postpartum	Pregnancy/ Postpartum	p-value	q-value
hexadecasphingosine (d16:1)*	0.95 ± 0.35	1.17 ± 0.41	0.87 ± 0.31	$3×10^{-4}$	$6×10^{-5}$
sphingosine 1-phosphate	1.19 ± 0.48	1.03 ± 0.33	1.22 ± 0.50	0.04	$5×10^{-3}$

[1] Pregnant and postpartum values represent median scaled peak areas. P values from paired Student's t-test. P-values, q-values, and fold change for all identified metabolites can be found in the supplementary files of our previous paper [9].

References

9. Enthoven LF, Shi Y, Fay EE, et al. The Effects of Pregnancy on Amino Acid Levels and Nitrogen Disposition. *Metabolites*. 2023;13(2):242. Published 2023 Feb 7. doi:10.3390/metabo13020242.
